# Chemoresistance induces enhanced adhesion and transendothelial penetration of neuroblastoma cells by down-regulating NCAM surface expression

**DOI:** 10.1186/1471-2407-6-294

**Published:** 2006-12-21

**Authors:** Roman A Blaheta, Frederick H Daher, Martin Michaelis, Christoph Hasenberg, Eva M Weich, Dietger Jonas, Rouslan Kotchetkov, Hans Willhelm Doerr, Jindrich Cinatl

**Affiliations:** 1 Zentrum der Chirurgie, Klinik für Urologie und Kinderurologie; Johann Wolfgang Goethe-Universität; Frankfurt am Main, Germany; 2 Zentrum der Hygiene, Institut für Medizinische Virologie; Johann Wolfgang Goethe-Universität; Frankfurt am Main, Germany

## Abstract

**Background:**

Drug resistance to chemotherapy is often associated with increased malignancy in neuroblastoma (NB). One explanation for the link between resistance and malignancy might be that resistance facilitates cancer progression and invasion. To investigate this hypothesis, adhesion, transendothelial penetration and NCAM (CD56) adhesion receptor expression of drug-resistant versus drug-sensitive NB tumor cells were evaluated.

**Methods:**

Acquired drug resistance was mimicked by exposing parental UKF-NB-2, UKF-NB-3 or IMR-32 tumor cells to increasing concentrations of vincristine- (VCR) or doxorubicin (DOX) to establish the resistant tumor cell sublines UKF-NB-2^VCR^, UKF-NB-2^DOX^, UKF-NB-3^VCR^, UKF-NB-3^DOX^, IMR-32^VCR ^and IMR-32^DOX^. Additionally, the malignant behaviour of UKF-NB-4, which already possessed the intrinsic multidrug resistance (MDR) phenotype, was analyzed. UKF-NB-4 exposed to VCR or DOX were designated UKF-NB-4^VCR ^or UKF-NB-4^DOX^. Combined phase contrast – reflection interference contrast microscopy was used to separately evaluate NB cell adhesion and penetration. NCAM was analyzed by flow cytometry, western blot and RT-PCR.

**Results:**

VCR and DOX resistant tumor sublines showed enhanced adhesion and penetration capacity, compared to their drug naïve controls. Strongest effects were seen with UKF-NB-2^VCR^, UKF-NB-3^VCR ^and IMR-32^DOX^. DOX or VCR treatment also evoked increased invasive behaviour of UKF-NB-4. The process of accelerated tumor invasion was accompanied by decreased NCAM surface and protein expression, and down-regulation of NCAM coding mRNA. Transfection of UKF-NB-4^VCR ^cells with NCAM cDNA led to a significant receptor up-regulation, paralleled by diminished adhesion to an endothelial cell monolayer.

**Conclusion:**

It is concluded that NB cells resistant to anticancer drugs acquire increased invasive capacity relative to non-resistant parental cells, and that enhanced invasion is caused by strong down-regulation of NCAM adhesion receptors.

## Background

Multiple-agent chemotherapy is the conventional therapy for patients with advanced stages of neuroblastoma (NB) and disseminated NB. However, drug resistance arises in the majority of stage IV and relapse NB disease, initially responding to chemotherapy [1]. Classical multidrug resistance is attributed to the elevated expression of ATP-dependent drug-efflux pumps such as P-glycoprotein (Pgp) or multidrug resistance-associated protein (MRP). Both drug-efflux pumps are thought to decrease cellular accumulation of anti-cancer drugs and therefore limit the success of chemotherapy regimens.

However, recent observations suggest that the hypothesis of drug-efflux driven chemoresistance might not always hold true. Retrospective analysis of bladder cancer patients submitted to chemotherapy have revealed no correlation between tumor progression and Pgp modification [2]. Overexpression of Pgp and MRP was also not correlated to chemoresistance and subsequent tumor recurrence and progression in sarcoma patients [3]. In particular, the clinical significance of the expression of the multidrug resistance gene (MDR1) product Pgp in NB is still a matter of debate. Although frequent expression of functional Pgp has been found in patients with NB, no correlation has been seen between Pgp, disease stage and histopathological grading [4]. Other studies have also failed to predict the correlation between MDR1 gene expression and clinical outcome for children with NB [5,6].

Recently, we developed multi-drug resistant NB in in vitro models and have demonstrated for the first time that an increased malignant phenotype in multidrug-resistant neuroblastoma cells is not mediated by up-regulated functional Pgp [7]. Rather, chemotherapy might have a direct impact on the biological characteristics of tumors leading to increased malignancy and metastasis [8]. Based on this, we hypothesized that chemoresistance itself might accelerate tumor progression and invasion.

To investigate the influence of chemotherapy on tumor invasion, we used an in vitro model of acquired drug resistance closely resembling progressive NB disease through long-term treatment of NB cell lines with vincristine- (VCR) or doxorubicin (DOX) to establish VCR and DOX-resistant tumor cell sublines (NB^VCR ^and NB^DOX^). We additionally analyzed NB tumor cells with intrinsic multidrug resistance (MDR) phenotype (UKF-NB-4) [9]. A transendothelial migration assay allowed the separate analysis of tumor cells which adhered to an endothelial cell monolayer and those which transmigrated beneath the endothelium. Since surface receptors are strongly involved in tumor invasion and data has indicated that changes in neural cell adhesion molecule (NCAM, CD56) expression play an essential part in the progression of NB, we investigated NCAM expression. This is of particular interest because the modulation of NCAM is a rate-limiting event in the metastatic dissemination of tumor cells [10].

The data provide evidence that NB cells resistant to anticancer drugs acquire increased invasive capacity relative to non-resistant parental cells, and that enhanced invasion is caused by strong down-regulation of NCAM adhesion receptors.

## Methods

### Cell cultures and induction of drug resistance

The human neuroblastoma cell lines UKF-NB-2, UKF-NB-3 and UKF-NB-4 were established in our laboratory from bone marrow metastases. IMR-32 was derived from LGC Promochem, Wesel, Germany. The vincristine- (VCR) and doxorubicin-resistant (DOX) cell sublines were established by exposing parental UKF-NB-2, UKF-NB-3 [8], or IMR-32 cells to increasing concentrations of the respective drug. Solutions of VCR (Sigma-Aldrich, Deisenhofen, Germany) and DOX (Farmitalia, Milan, Italy) were prepared in accordance to the manufacturer's instructions. The initial VCR and DOX concentrations were 0.2 ng/ml and 2 ng/ml medium, respectively. The resistant sublines were grown for more than 6 months in Iscove's modified Dulbecco's medium (IMDM; Gibco, Karlsruhe, Germany) supplemented with 10% fetal calf serum (FCS, Gibco) and 10 ng/ml VCR (UKF-NB-2^VCR^, UKF-NB-3^VCR^, IMR-32^VCR^) or 20 ng/ml DOX (UKF-NB-2^DOX^, UKF-NB-3^DOX^, IMR-32^DOX^). All doses were in the range of clinical plasma concentrations. Multidrug resistant parental UKF-NB-4 cells were exposed to 50 ng/ml VCR (UKF-NB-4^VCR^) or 100 ng/ml DOX (UKF-NB-4^DOX^). All cells were subcultured at 5-day intervals.

Human endothelial cells (HUVEC) were isolated from human umbilical veins and harvested by enzymatic treatment with chymotrypsin. HUVEC were grown in Medium 199 (M199; Biozol, Munich, Germany), supplemented with 10% FCS, 10% pooled human serum, 20 μg/ml endothelial cell growth factor (Boehringer, Mannheim, Germany), 0.1% heparin, 100 ng/ml gentamycin and 20 mM HEPES-buffer (pH 7.4). Subcultures from passages 2–6 with intact morphology and proliferative capacity were selected for experimental use.

### Monolayer invasion assay

Round cover slips were treated with 3-aminopropyl-triethoxy-silan (2%; Sigma, München, Germany) – acetone solution for 60 minutes (20°C) to allow firm adhesion of HUVEC and placed into six-well multiplates (Falcon Primaria; Becton Dickinson, Heidelberg, Germany). HUVEC subcultures were transferred to prepared multiplates in complete HUVEC-medium. When confluency was reached, 0.5 × 10^6 ^neuroblastoma cells/well were carefully added to the HUVEC monolayer for various time periods. Subsequently, non-adherent neuroblastoma cells were washed off using warmed (37°C) M199. The remaining cells were fixed with 1% glutaraldehyde. Bound tumor cells were counted in five different fields (5 × 0.25 mm^2^) using a phase contrast microscope (20 × objective) and the mean cellular adhesion rate was calculated.

To separately assess neuroblastoma cells which had penetrated the HUVEC monolayer, a reflection interference contrast microscope with a Ploem apparatus was used. The relevant theoretical evaluation of reflection contrast microscopy is given by Bereiter-Hahn et al. and Gingell and Todd [11,12]. The resulting images were visualized and amplified by a Proxitronic CCD-camera (Proxitronic, Bensheim, Germany). The number of penetrated neuroblastoma cells were then quantified using the image analyzing system ARGUS 20 (Hamamatsu, Hersching, Germany). To optimize the signal to noise ratio, online background subtraction and averaging of 8 images was performed, using the image processing system QUANTIMET Q520 (Cambridge Instruments, Bensheim). Cells that penetrated the HUVEC monolayer were counted in five different fields (5 × 0.25 mm^2^, 20 × objective), and mean penetration rate was calculated.

### Cell proliferation

Proliferative activity of NB cells and HUVEC was estimated by the PicoGreen assay as described elsewhere [13]. Briefly, at several time points after plating the cells in six-well multiplates, culture medium was removed and cells were digested with papain (0.125 mg protein/ml) for 20 h at 60°C. Thereafter, the fluorescent dye PicoGreen (MoBiTec, Goettingen, Germany), which shows high specificity for dsDNA, was added (1:200 dilution) for 10 min at 20°C. Fluorescence intensity was determined using a computer-controlled fluorescence reader (Cytofluor 2300 plate scanner; Millipore, Eschborn, Germany) at λex = 485 nm and λem = 530 nm.

### Plasmids and transfection

UKF-NB-4^VCR ^cells were transfected using the calcium phosphate co-precipitation method with full-length cDNA, encoding the human NCAM-140 kDa isoform inserted in sense of the eukaryotic expression vector pHβ-Apr-1-neo [14]. Control cells were transfected with the expression vector alone. 0.5 × 10^6 ^NB were seeded in 25 cm^2 ^culture flasks, grown overnight, and transfected with 2 μg of DNA. After culturing for 16 h in complete medium, cells were washed twice with PBS without Ca^2+ ^and Mg^2+ ^and then incubated with fresh cell culture medium. Efficiency of NCAM transfection using pHβ-Apr1-neo was investigated fluorometrically by calcium phosphate co-precipitation of pHβ-Apr1-neo in combination with pEGFP-N1 vector (Clontech GmbH, Heidelberg, Germany) at a ratio of 1:1. pEGFP-N1 encodes a red-shifted variant of wild-type GFP which has been optimized for brighter fluorescence and higher expression in mammalian cells. Fluorescence analysis revealed a transfection rate of pHβ-Apr1-neo of 30%. Viability of the cells, which was controlled by trypan blue dye exclusion or by quantitative fluorescence analysis of propidium iodide uptake, was > 90%.

### Evaluation of NCAM surface expression

Neuroblastoma cells were disaggregated mechanically, washed in blocking solution (PBS, 0.5% BSA) and then incubated for 60 min at 4°C with FITC-conjugated monoclonal antibody anti-CD56 which detects the NCAM 120, 140 and 180 kDa isoform (clone 16.2). NCAM expression of NB cells was then measured using a FACscan (Becton Dickinson; FL-1H (log) channel histogram analysis; 1 × 10^4 ^cells/scan) and expressed as mean fluorescence units (MFU). A mouse IgG1-FITC was used as the isotype control.

To explore NCAM localization, tumor cells were transferred to round cover slips which were placed in a 24 well multiplate. Upon reaching confluency, cell cultures were washed two times with PBS (with Ca^2+ ^and Mg^2+^) and then fixed in cold (-20°C) methanol/acetone (60/40 v/v). Subsequently, cells were washed again with PBS (without Ca^2+ ^and Mg^2+^), and afterwards once with blocking buffer (0.5% BSA in PBS without Ca^2+ ^and Mg^2+^). After removing the washing buffer, cells were incubated for 60 min with FITC-conjugated anti-NCAM monoclonal antibody. To prevent photobleaching of the fluorescent dye, cover glasses with stained cells were taken out of the wells and the residual liquid was removed. The cells were then embedded in an antifade reagent/mounting medium mixture (ProLong™ Antifade Kit, MoBiTec, Göttingen, Germany) and mounted on slides. The slides were viewed using a confocal laser scanning microscope (LSM 10; Zeiss, Jena, Germany) with a plan-neofluar × 100/1.3 oil immersion objective.

### Western blot analysis

Total NCAM content in neuroblastoma cells was evaluated by Western blot analysis: cell lysates were applied to a 7 % polyacrylamide gel and electrophoresed for 90 min at 60 V. The protein was then transferred to nitrocellulose membranes. After blocking, the membranes were incubated overnight with the anti-NCAM-antibody (dilution 1:1000). HRP-conjugated goat-anti-mouse IgG (Upstate Biotechnology, Lake Placid, NY, USA; dilution 1:5000) served as the secondary antibody. The membrane was briefly incubated with ECL detection reagent (ECL™, Amersham) to visualize the proteins and exposed to an x-ray-film (Hyperfilm™ EC™, Amersham).

### Reverse transcription/polymerase chain reaction (RT/PCR)

Total RNA was extracted and purified with Trizol reagent according to the manufacturer's instructions and treated with RNase-free DNase. The NCAM primer sequences were as follows: for NCAM-180 (AccNo AK056258): 5'CGAGGCTGCCTCCGTCAGCACC 3' and 5'CCGGATCCATCATGCTTTGCTCTCG 3'; for NCAM-140 (AccNo U63041): 5'GAACCTGATCAAGCAGGATGACGG 3' and 5'CCGGATCCATCATGCTTTGCTCTCG 3' [15]. Internal controls for the RT-PCR reaction was performed by running parallel reaction mixtures with the housekeeping gene GAPDH: 5'ATCTTCCAGGAGCGAGATCC 3' and 5'ACCACTGACACGTTGGCAGT 3'. RNA (1–10 μg) was reverse transcribed and the resulting cDNA directly added to the polymerase chain reaction. Amplification reactions (20 μl) were performed in the presence of 1/10 (2 μl) of the cDNA reaction, with an initial incubation step at 94°C for 1 min. Cycling conditions consisted of denaturation at 94°C for 1 min, annealing at 55°C for 1 min, and extension at 72°C for 1 min over a total of 30 cycles. The reaction was completed by another incubation step at 72°C for 10 min. The PCR products were subjected to electrophoresis in 2% agarose gel and visualized by ethidium bromide.

### Statistics

All experiments were performed 3–6 times. Statistical significance was investigated by the Wilcoxon–Mann-Whitney-U-test. Differences were considered statistically significant at a p value less than 0.05.

## Results

### Drug resistance caused elevated tumor cell adhesion and transendothelial penetration

**Figure 1:**
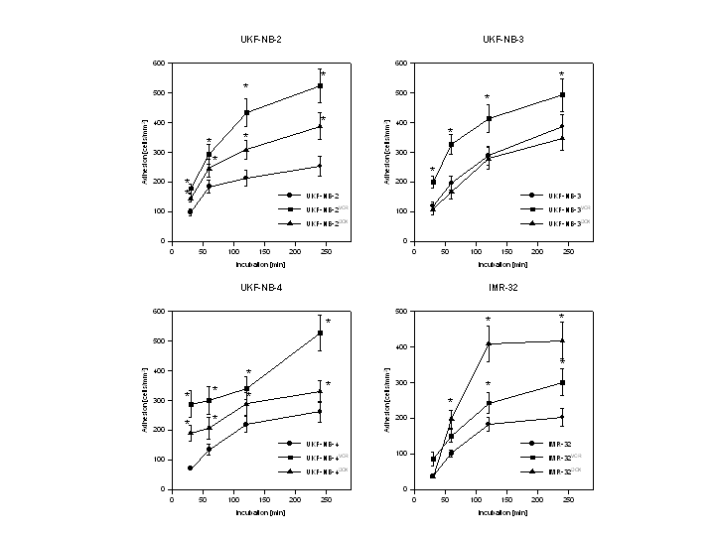
Adhesion kinetics of parental control cells (UKF-NB-2, UKF-NB-3, UKF-NB-4, IMR-32) versus vincristine (UKF-NB-2^VCR^, UKF-NB-3^VCR^, UKF-NB-4^VCR^, IMR-32^VCR^) or doxorubicin resistant subpopulations (UKF-NB-2^DOX^, UKF-NB-3^DOX^, UKF-NB-4^DOX^, IMR-32^DOX^). Adhesion capacity is depicted as tumor cell adhesion/mm^2 ^(mean ± SD; n = 6). X-axis indicates the period of co-culture. *indicates significant difference to the controls.

Both VCR and DOX resistance was accompanied by elevated tumor cell adhesion to HUVEC. Table 1 indicates the level of drug resistance and figure 1 shows adhesion kinetics of control versus VCR or DOX resistant cell lines. The number of adherent parental tumor cells rapidly increased and reached a plateau after 60 (UKF-NB-2) or 120 min (UKF-NB-4, IMR-32). UKF-NB-3 adhesion steadily increased up to 240 min. Significantly more UKF-NB-2^VCR ^and UKF-NB-3^VCR ^cells attached to HUVEC, compared to the drug naïve control cells. Only slight differences were observed between IMR-32^VCR ^and IMR-32 controls (not significant at 30 and 60 min). Surprisingly, adhesion events of UKF-NB-4 possessing the MDR phenotype further increased when the parental cells were exposed to VCR. The same was true when DOX was applied to UKF-NB-4, although UKF-NB4^VCR ^had a higher adhesion capacity than UKF-NB^DOX^.

DOX resistance caused rapid and strong up-regulation of IMR-32 adhesion. DOX resistance was also accompanied by elevated adhesion of UKF-NB-2 cells, when compared to the controls. However, this effect was not as intense as in UKF-NB-2^VCR^cells. No significant difference was observed between adhesion characteristics of UKF-NB-3^DOX ^and UKF-NB-3 controls.

**Figure 2:**
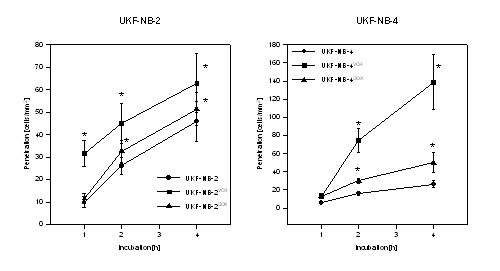
Penetration kinetics of parental control cells (UKF-NB-2, UKF-NB-4) versus vincristine (UKF-NB-2^VCR^, UKF-NB-4^VCR^) or doxorubicin resistant subpopulations (UKF-NB-2^DOX^, UKF-NB-4^DOX^). To identify transmigrated NB from the cells which had bound to HUVEC, a reflection interference contrast microscope with a Ploem apparatus was used (see materials and methods for details). X-axis indicates the period of co-culture. *indicates significant difference to the controls.

Tumor cell penetration through HUVEC monolayers was evaluated on UKF-NB-2 and the multidrug resistant UKF-NB-4 cells. All resistant cell sublines had significantly higher number of transmigrated cells compared to the parental cell lines: +70% after 2 h, +38% after 4 h for UKF-NB-2^VCR^, +362% after 2 h, + 430% after 4 h for UKF-NB-4^VCR^, +23% after 2 h, +13% after 4 h for UKF-NB-2^DOX^, +81% after 2 h, +92% after 4 h for UKF-NB-4^DOX ^cell sublines (figure 2). 4 h transmigration was also evaluated on IMR-32^DOX ^or IMR-32^VCR ^cells. Both sublines showed significantly higher penetration capacity than the parental controls (IMR-32^DOX^: +294% mean; IMR-32^VCR^: +26% mean).

The PicoGreen assay, which had been carried out as well, revealed no proliferative activity of tumor cells during the experiment which might account for enhanced tumor adhesion and penetration.

### NCAM was down-regulated in drug-resistant cell lines

In good accordance to the adhesion pattern, NCAM surface expression was reduced on VCR-resistant cells: strongly on UKF-NB-2^VCR^, UKF-NB-3^VCR ^or UKF-NB-4^VCR ^cells, and slightly on IMR-32^VCR ^cells, compared to the NCAM level on the parental cell lines.

**Figure 3:**
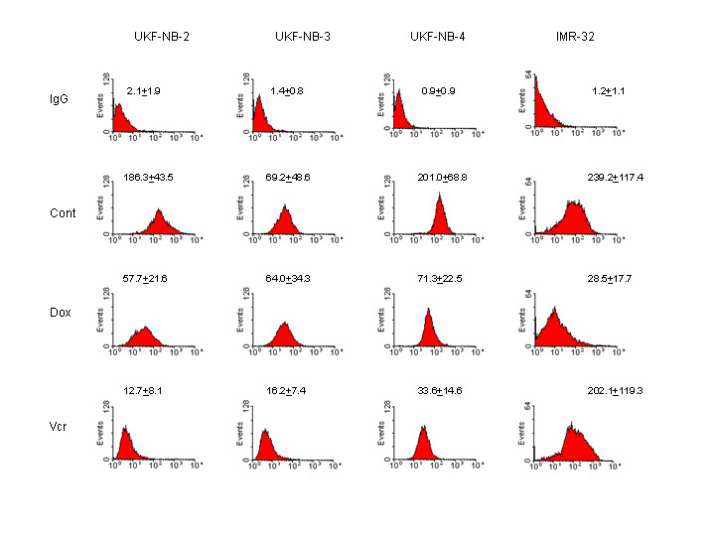
NCAM surface expression on UKF-NB-2, UKF-NB-3, UKF-NB-4, IMR-32 controls and the respective doxorubicin- (DOX) or vincristine resistant sublines (VCR). An FITC-conjugated monoclonal antibody anti- CD56, clone 16.2, was used to detect the NCAM 120, 140 and 180 kDa isoform. A mouse IgG1-FITC served as the isotype control (IgG). The figure shows the histogram analysis of one representative experiment. The complete experimental data set (MFU +/- SD; n = 6) is given above the histograms.

DOX-resistant cells showed a spectrum of decrease of NCAM surface receptors: distinct loss in IMR-32^DOX ^cells, moderate reduction in UKF-NB-2^DOX ^or UKF-NB-4^DOX ^cells, and slight, non-significant decrease on UKF-NB-3^DOX ^cells. Figure 3 shows the histogram analysis of one representative experiment.

**Figure 4:**
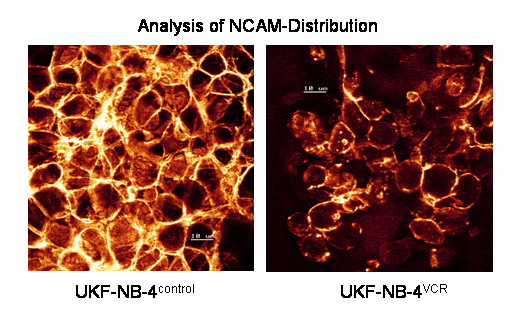
Confocal analysis of NCAM distribution. UKF-NB-4 control versus UKF-NB-4^VCR ^tumor cells were grown in standard medium. FITC conjugated monoclonal antibody clone 16.2 was used to analyze NCAM. The figure shows distinct NCAM expression at the intercellular boundaries on UKF-NB-4 control cells which is reduced in UKF-NB-4^VCR^. Scale = 10 μM. × 100/1.3 oil immersion objective.

NCAM localization was analyzed on UKF-NB-4 cells. Confocal microscopy demonstrated intense and homogenous NCAM accumulation along the cell boundaries of UKF-NB-4 cells. When UKF-NB-4 cells were exposed to VCR (UKF-NB-4^VCR^), NCAM partially disappeared from the cell surface and the tumor aggregates became smaller by cell disaggregation (figure 4). As VCR treatment did not induce proliferation blockade, diminishing the tumor aggregates was not caused by a reduced cell growth rate.

**Figure 5:**
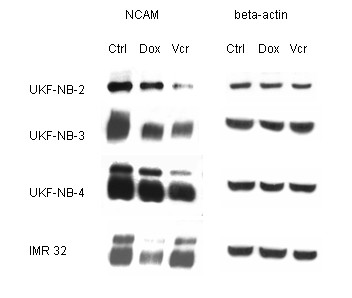
Western blot analysis of NCAM from the proteins of UKF-NB-3, UKF-NB-4, IMR-32 (Ctrl) versus vincristine (VCR) or doxorubicin resistant subpopulations (DOX). Cell lysates were incubated with anti NCAM (clone 16.2) monoclonal antibodies, which detect the NCAM 120, 140 and 180 kDa isoform. beta-actin served as the internal control. The figure shows one representative from three separate experiments.

Western blot experiments were carried out to measure the intracellular NCAM protein content in drug resistant versus non-resistant tumor cells. Figure 5 shows reduced NCAM proteins in UKF-NB-3^VCR ^and UKF-NB-4^VCR ^cells, compared to the controls, but similar NCAM content in IMR-32^VCR ^and IMR-32 control cells. Based on DOX resistant cell lines, NCAM proteins were strongly diminished in IMR-32^DOX^, and slightly reduced in UKF-NB-4^DOX^. Remarkably, NCAM amount was down-modulated similarly in UKF-NB-3^DOX ^and UKF-NB-3^VCR^, a phenomenon which was not observed on the cell surface (NCAM^control ^> NCAM^DOX ^> NCAM^VCR^; flow cytometry analysis; figure 3).

**Figure 6:**
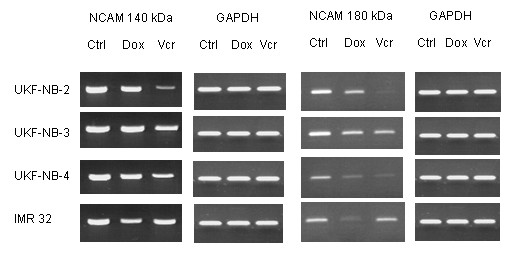
Semi quantitative RT-PCR analysis of NCAM 140 kDa and 180 kDa RNA in parental UKF-NB-2, UKF-NB-3, UKF-NB-4, IMR-32 (Ctrl) versus vincristine (VCR) or doxorubicin resistant subpopulations (DOX). RNA were extracted, reverse-transcribed, and submitted to semiquantitative reverse transcription-PCR using gene specific primers as indicated in materials and methods. The internal control for the RT-PCR reaction was performed by running parallel reaction mixtures with the housekeeping gene GAPDH. The figure shows one representative from three separate experiments.

A further assessment was carrid out to determine whether NCAM mRNA expression was affected by VCR or DOX. Concerning VCR resistance, mRNA encoding the 140 kDa NCAM isoform was down-regulated in the following order: UKF-NB-2^VCR ^> UKF-NB-4^VCR ^> UKF-NB-3^VCR ^(figure 6). 140 kDa NCAM mRNA was also reduced in IMR-32^DOX ^(strongly) and UKF-NB-2^DOX ^or UKF-NB-4^DOX ^(moderately). mRNA encoding the 180 kDa isoform was also detected in all cell lines, bands of which disappeared in UKF-NB-2^VCR^, UKF-NB-4^VCR^, and IMR-32^DOX^, or were diminished in UKF-NB-2^DOX^, UKF-NB-3^DOX ^(as well as in UKF-NB-3^VCR^), and UKF-NB-4^DOX ^cells (figure 6).

### NCAM transfection reduced NB cell adhesion

**Figure 7:**
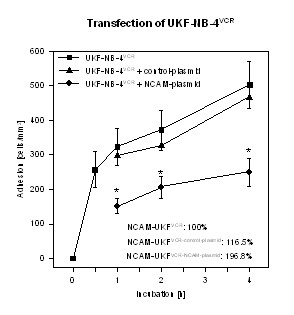
Adhesion kinetics of UKF-NB-4^VCR ^and of UKF-NB-4^VCR ^transfected with 2 μg of full-length cDNA encoding the human NCAM-140 kDa isoforms are shown. Control cells were transfected with the expression vector alone. Data on NCAM surface expression, evaluated by flow cytometry, are given in the lower right hand corner. 100% value = 51.4 ± 18.7 MFU. X-axis indicates the period of co-culture (mean ± SD, n=3). *indicates significant difference to UKF-NB-4^VCR^.

The relevance of NCAM for tumor cell binding to HUVEC was demonstrated by transfection experiments. Transfection of UKF-NB-4^VCR ^cells with NCAM cDNA reverted the VCR evoked down-regulating effect on this receptor and resulted in a two-fold enhancement of NCAM on the cellular membrane. Viability of UKF-NB-4^VCR ^was not affected by this procedure. Adhesion of transfected UKF-NB-4^VCR ^to HUVEC was significantly reduced, compared to non-transfected cells (figure 7). Transfection of UKF-NB-4^VCR ^with a control plasmid did not change NCAM expression and tumor cell adhesion.

## Discussion

Intrinsic or acquired resistance to anticancer agents is a major obstacle to the success of chemotherapy. In the present work, we compared the invasive properties of drug sensitive NB with their drug-resistant sublines, as well as intrinsically resistant NB which were further treated with VCR or DOX. The selection of the various tumor sublines mimicked the severe problems arising in patients during chemotherapy. Based on our model, we demonstrate that drug resistant cancer cells develop an increased malignant phenotype, in part due to enhanced adhesion and transendothelial penetration, accompanied by a significant down-regulation of the adhesion receptor NCAM.

Our findings might explain the paradoxical situation that tumors sometimes become progressively more malignant during the course of chemotherapeutic treatment. Slotman et al. found an increased incidence of hematogenous metastases with induction chemotherapy for patients with advanced head-and-neck squamous carcinomas, compared to patients receiving only surgery and radiotherapy. They also found an increase in metastases at sites other than the usual pulmonary site [16]. Stefani and coworkers reported that the use of hydroxyurea in combination with radiotherapy for the treatment of head and-neck cancer yielded no benefit in terms of response rate or survival, but rather increased the incidence of distant metastases from 8% to 23% [17]. The administration of a single drug dose may be sufficient to induce spontaneous metastasis [18].

Two reports have been published recently which underline our observations and suggest that drug resistance in melanoma and carcinoma cells may confer to a more invasive phenotype [19,20]. Nevertheless, it is still hypothesized that resistance to cytotoxic agents and invasive potential is associated with the over-expression of Pgp drug transporters [19,21]. Notably, increased Pgp expression was found in metastatic NB cells from bone marrow samples, as compared to non-metastatic NB [22]. Previous experiments on UKF-NB-2 and UKF-NB-3 cells also revealed high amounts of functionally active Pgp in both DOX and VCR resistant sublines. However, expression of Pgp was 3.5-fold higher in UKF-NB-2^DOX ^when compared to UKF-NB-2^VCR ^cells, and therefore does not correlate to the changes seen with respect to cell adhesion and transendothelial penetration (UKF-NB-2^DOX ^< UKF-NB-2^VCR^). Furthermore, pharmacological blockage of Pgp in resistant UKF-NB-2 and UKF-NB-3 cell lines restored chemosensitivity, but had no effect on colony formation and cell survival under serum free conditions [7,8].

Therefore, up-regulation of Pgp itself probably does not play a leading role in increasing NB malignant biology. However, concomitant deregulation of other pathways may result in increased metastatic potential, thus leading to a poorer prognosis of advanced forms of NB disease. In our cell culture model, modulation of NCAM seems to be the major parameter responsible for altered cell adhesion and penetration. This assumption is based on three observations: 1. Enhanced tumor binding and transmigration of drug resistant cell populations only occured when NCAM receptor expression was reduced. 2. NCAM loss strongly correlated with the elevation of adhesion and penetration capacity, and 3. Transfection of UKF-NB-4^VCR ^cells with NCAM cDNA up-regulated the NCAM expression level and diminished the interaction events between tumor and endothelial cells. In good accordance to the present data, we recently demonstrated an inverse correlation between NCAM expression and NB cell adhesion, assessed on 11 NB cell lines. In particular, transfection with a cDNA encoding the human NCAM-140 kD isoform enhanced NCAM expression and diminished initial NB cell adhesion, treatment with NCAM antisense oligonucleotides reduced NCAM surface level and induced up-regulation of NB cell adhesion to endothelium [10].

Several studies have demonstrated the important role of NCAM in tumor migration and metastasis, with an inverse relationship between migratory potential of the cells and NCAM expression. In primitive neuroectodermal tumor cells, an increase in NCAM was paralleled by a significant reduction in cellular motility and adhesion capacity [23,24]. In a rat model, NCAM-transfected glioma tumor cells became less invasive and destructive than control cells with a low NCAM expression level [25]. Diminished expression of NCAM was also associated with clinically aggressive colon cancers [26-28], and dissemination of pancreatic beta tumor cells [29,30]. Tezel et al. suggested that NCAM expression in tubular adenocarcinoma of the pancreas has a significant impact on overall patient survival [31]. It is currently assumed that NCAM, in its function as a homophilic receptor, stabilizes the primary tumor or tumor cell aggregates, while circulating in the blood vessels. Reduction of the NCAM expression level might lead to a reduction in cell-cell binding forces, and hence to the release of tumors as single cells. The less NCAM, the more metastatic cells leave the tumor mass, and the more penetration events can take place [10]. Consequently, NCAM loss observed in DOX and VCR resistant NB tumor cells allows more cells to transmigrate the endothelial blood barrier and extravasate into surrounding tissue. In line with this, establishment of DOX or VCR resistant human glioma cells was accompanied by NCAM reduction and a concomitant down-regulation of adhesiveness [32]. Analyses of tumor samples from relapsed patients have to be carried out to strenghthen the relevance of the in vitro findings.

Recently, it has been documented that the loss of NCAM function causes the formation of lymph node metastasis in pancreatic beta cell carcinogenesis via vascular endothelial growth factor -C and -D-mediated lymphangiogenesis [33]. In a previous work, loss of NCAM resulted in a failure to activate beta1 integrins via fibroblast growth factor receptor signalling [29]. Both findings implicate that NCAM, beside its role as an aggregation stabilizer, may also act in terms of a "metastasis suppressor protein", controlling proteins and genes that specifically inhibit the ability of tumor cells from forming metastases [34].

Our hypothesis of drug evoked alterations of NB tumor cell binding and transvasation also appears to be applicable to tumor cells with intrinsic drug resistance. UKF–NB-4 cells with a multidrug resistant phenotype retained the potential to become even more aggressive when treated with DOX or VCR. From a clinical viewpoint, chemotherapy of NB might induce adverse effects concerning tumor biology. In fact, single chemotherapy may amplify the process of tumor progression if drug-resistant tumor subclones are present in the initial tumor burden.

The finding that extended drug exposure contributes to distinct modifications of adhesion receptors and invasive potential of single cancer cells, independent of Pgp over-expression, may be a signal to re-assess current therapeutic protocols. In fact, the use of escalating doses of cyclophosphamide and prolonging conventional chemotherapy with the same drugs has failed to improve the metastasis complete response rate in NB patients [35]. Since initial pharmacological attempts to inhibit drug efflux and increase intracellular drug concentrations have not provided the desired clinical benefit in relapsed or resistant paediatric cancers [36], the consequences of drug resistance might preferably be treated by agents which reverse cellular adhesion. In this context, the branched-chain fatty acid valproate has been demonstrated to inhibit tumor cell motility and adhesion by up-regulating NCAM [37,38]. The same is true for the differentiation inducing compound trichostatin A [39]. A significant increase of NCAM expression level accompanied by blocking cellular adhesion has also been observed when neuroblastoma cells were treated with retinoic acid [40,41]. The introduction of differentiation inducing drugs may therefore advance the treatment of relapsed tumors. Currently, several studies dealing with this issue are underway.

It is important to note that extended drug exposure evoked different responses in the in vitro system described here. Although drug resistance was established in all tumor sublines, VCR resistance led to increased attachment of UKF-NB-2, UKF-NB-3 or UKF-NB-4 cell lines, but not of IMR-32, whereas DOX resistance induced marked elevation of IMR-32 attachment but had no influence on UKF-NB-3 adhesion. Alterations in the NCAM expression level followed the same pattern. Therefore, modifications of the invasive program cannot be expected in every case where drug resistance in tumor cells develops. VCR or DOX resistance might convert NCAM triggered cell adhesion in some but not in all tumor subpopulations, or in some but not in all tumor patients where chemotherapy has failed. It is still unknown how drug resistant tumor cells are selected to became "responders", i.e. increase their invasive activity. However, if our hypothesis holds true, a critical selection of those "responding" patients should be carried out who might be predestined for innovative antitumoral, adhesion blocking strategies.

## Conclusion

Evidence is presented showing that tumor cells with acquired resistance to chemotherapeutic drugs have enhanced invasive potential caused by a strong down-regulation of NCAM adhesion receptors. However, the data are limited to DOX and VCR resistant NB cell lines. Therefore, the hypothetical possibility that treatment with chemotherapeutic drugs in general may promote cancer invasion and metastasis needs further investigation. There is also no doubt that adhesion receptors different from NCAM may be altered during anti-tumor therapy. This includes E-cadherin [42,43], as well as CD44 or ICAM-1 [19,44,45]. Presumably, determination of the expression pattern of a cohort of surface receptors in individual cancer cells will allow better prediction of the clinical response to chemotherapy. Detailed knowledge of receptor driven tumor dissemination may also provide new ways to overcome drug resistance.

## Competing interests

The author(s) declare that they have no competing interests.

## Authors' contributions

RAB performed adhesion and penetration studies and drafted the manuscript. FHD carried out flow cytometry, confocal microscopy and RT-PCR. MM performed analysis of IC50 values. CH and EV performed western blotting. DJ participated in the conception and design of the study. RK contributed to the manuscript design, data analysis and finalisation. HWD and JC participated in the conception and design of the study and its coordination. All authors read and approved the final manuscript.

## Pre-publication history

The pre-publication history for this paper can be accessed here:



## Figures and Tables

**Table 1:** Level of drug resistance, indicated as IC_50_-values (ng/ml)

Cell line	IC_50_-doxorubicin	IC_50_-vincristin
UKF-NB-2	7.4 ± 3.8	0.78 ± 0.23
UKF-NB-2^DOX^	62 ± 12	22 ± 7
UKF-NB-2^VCR^	91 ± 18	51 ± 12
		
UKF-NB-3	8.9 ± 2.0	0.37 ± 0.12
UKF-NB-3^DOX^	63 ± 11	195 ± 41
UKF-NB-3^VCR^	91 ± 18	51 ± 12
		
IMR-32	6.2 ± 3	0.73 ± 0.51
IMR-32^DOX^	75 ± 14	2.08 ± 0.34
IMR-32^VCR^	43 ± 11	20 ± 6
		
UKF-NB-4	214 ± 31	26 ± 8
